# QCL Infrared Spectroscopy Combined with Machine Learning as a Useful Tool for Classifying Acetaminophen Tablets by Brand

**DOI:** 10.3390/molecules29153562

**Published:** 2024-07-28

**Authors:** José A. Martínez-Trespalacios, Daniel E. Polo-Herrera, Tamara Y. Félix-Massa, Samuel P. Hernandez-Rivera, Joaquín Hernandez-Fernandez, Fredy Colpas-Castillo, John R. Castro-Suarez

**Affiliations:** 1 Mechanical Engineering Program, School of Engineering, Universidad Tecnológica de Bolívar, Parque Industrial y Tecnológico Carlos Vélez Pombo, Cartagena 130001, Colombia; jmartinezt@utb.edu.co (J.A.M.-T.); jhernandezf@unicartagena.edu.co (J.H.-F.); 2 Chemistry Program, Department of Natural and Exact Sciences, San Pablo Campus, University of Cartagena, Cartagena 130015, Colombia; dpoloh1@unicartagena.edu.co (D.E.P.-H.); fcolpasc1@unicartagena.edu.co (F.C.-C.); 3 Center for Chemical Sensors and Chemical Imaging and Surface Analysis Center, Department of Chemistry, University of Puerto Rico, Mayaguez, PR 00681, USA; tamara.felix@upr.edu (T.Y.F.-M.); samuel.hernandez3@upr.edu (S.P.H.-R.); 4 Department of Natural and Exact Science, Universidad de la Costa, Barranquilla 080002, Colombia; 5 Área Básicas Exactas, Universidad del Sinú, Seccional Cartagena, Cartagena 130015, Colombia

**Keywords:** vibrational spectroscopy, machine learning, counterfeit drugs, chemometrics, mid-infrared

## Abstract

The development of new methods of identification of active pharmaceutical ingredients (API) is a subject of paramount importance for research centers, the pharmaceutical industry, and law enforcement agencies. Here, a system for identifying and classifying pharmaceutical tablets containing acetaminophen (AAP) by brand has been developed. In total, 15 tablets of 11 brands for a total of 165 samples were analyzed. Mid-infrared vibrational spectroscopy with multivariate analysis was employed. Quantum cascade lasers (QCLs) were used as mid-infrared sources. IR spectra in the spectral range 980–1600 cm^−1^ were recorded. Five different classification methods were used. First, a spectral search through correlation indices. Second, machine learning algorithms such as principal component analysis (PCA), support vector classification (SVC), decision tree classifier (DTC), and artificial neural network (ANN) were employed to classify tablets by brands. SNV and first derivative were used as preprocessing to improve the spectral information. Precision, recall, specificity, F1-score, and accuracy were used as criteria to evaluate the best SVC, DEE, and ANN classification models obtained. The IR spectra of the tablets show characteristic vibrational signals of AAP and other APIs present. Spectral classification by spectral search and PCA showed limitations in differentiating between brands, particularly for tablets containing AAP as the only API. Machine learning models, specifically SVC, achieved high accuracy in classifying AAP tablets according to their brand, even for brands containing only AAP.

## 1. Introduction

The detection of chemical substances in different solid, liquid, or gaseous matrices has always been of interest to research groups. These substances of interest may be active pharmaceutical ingredients (APIs) in pharmaceutical formulations that, if misused, could harm people. Recently, the United States Food and Drug Administration (US FDA) and the World Health Organization (WHO) have raised the alarm about the increase in counterfeit drugs in the world that are being distributed in the market as legal drugs, with over-the-counter drugs being the most susceptible to this drug trafficking. These counterfeit drugs may contain no active ingredient, be in poor condition, or be contaminated. However, these drugs may have the right active ingredient but the wrong dosage. Whatever the case, these counterfeit drugs are illegal and can be harmful to your health [1,2].

In 2017, the WHO estimated that 10.5% of medications worldwide are either subpar or fake. As per a report, the incidence of counterfeit medicines was around 13.6% in low- and middle-income countries (LMICs) [3]. Asia, specifically India, leads in the production of counterfeit medicines, with approximately 35–75% of fake medicines being produced in India. However, as per the reports of the Pharmaceutical Security Institute (PSI), a not-for-profit membership organization, the maximum percentage of counterfeit drugs seized was higher in North America, followed by Asia–Pacific [2,4]. Developing nations are home to a sizable part of the counterfeit drugs being circulated in the world. As per the data from WHO, every 1 in 10 medicinal products in developing countries is counterfeit or spurious [5].

Counterfeiting is not limited to vaccines alone. Counterfeited versions of essential items needed during the COVID-19 pandemic, such as face masks, PPE kits, N95 masks, gloves, sanitizers, and diagnostic kits, along with medicines such as antivirals, chloroquine, paracetamol, and vitamin C, were also abundant in the market [6]. The illegal market for drugs increased by more than 400% at the end of 2021 [7].

Acetaminophen (AAP), or paracetamol, is one of the most widely used active ingredients in pharmaceutical formulations. AAP is commonly used as an analgesic and antipyretic to relieve pain and reduce fever. It is specifically prescribed to treat various conditions such as headaches, muscle aches, arthritis, back pain, toothaches, colds, and fevers. During COVID-19, the volume of requests for paracetamol increased by more than 110%. Thus, the indication of its use as an anti-inflammatory drug, the lack of a prescription, and the ease in procuring it led, in some cases, to lethal consequences. AAP overdose remains the leading cause of death or transplantation due to acute liver failure in many parts of the world. The amount supplied to the patient is of utmost importance; an insufficient amount could prolong the treatment of a given condition and, even worse, too much could have adverse consequences for the patient’s health. Due to its easy availability without prescription, deliberate consumption or overdosage can happen. Overdoses of AAP are associated with liver toxicity and renal failure. Liver toxicity begins with AAP plasma levels in the range of 120 µgmL^−1^ 4 h after ingestion, and severe damage occurs with plasma levels up to 200 µgmL^−1^ 4 h after ingestion [8,9].

Many analytical methods have been used and proposed by the scientific community for the determination of AAP, both for pharmaceutical formulations and biological samples. Among them, chromatographic [10,11] and optical (UV-VIS) [12,13] methods are the most widely used. Other analytical methods, such as electrochemical [14,15], have been recently reported by the scientific community. These techniques are efficient in terms of reproducibility and low detection limits. However, in their chemical analysis process, sample preparation steps are carried out, lengthening the analysis time and making their use in the field difficult.

Vibrational spectroscopic techniques such as Raman spectroscopy and infrared spectroscopy have been frequently used for the analysis of active ingredients in pharmaceutical products [16,17]. Within the infrared, near-infrared spectroscopy has been widely used in pharmaceutical applications [18,19], especially for the determination of AAP in solid and liquid pharmaceutical formulations [20].

Some contributions that have made use of vibrational spectroscopy for the analysis of AAP are R. Szostak et al. [21] who proposed the determination of acetylsalicylic acid and acetaminophen in pharmaceuticals by partial least squares (PLS) and principal component regression (PCR) treatment of Fourier transform (FT)–Raman spectroscopic data. The proposed method was tested on powdered samples. The relative standard error of predictions (RSEPs) was calculated for both calibration and prediction datasets. These values ranged from 0.7% to 2.0% for the calibration set and from 0.8% to 2.3% for the prediction set across the different PLS models.

M. Khanmohammadi et al. [22] carried out a spectroscopy method based on Fourier transform near-infrared (FT-NIR) spectroscopy and chemometric techniques to classify paracetamol preparations according to polymorphic changes. These studies were carried out on standard samples and paracetamol preparations (acetaminophen tablets). M. Mallah et al. [23] developed a method for the quantification of paracetamol in solid formulations using transmission FTIR spectroscopy with KBr pellets. The spectral region of 1800–1000 cm^−1^ was utilized for the quantification of paracetamol content, achieving a regression coefficient (R^2^) of 0.999. The limits of detection and quantification using FTIR spectroscopy were 0.005 mg g^−1^ and 0.018 mg g^−1^, respectively. O. O. Oloyede. et al. [24] highlighted a screening methodology through the combination of spectroscopic (Raman/FTIR) and X-ray diffraction techniques with PCA as a predictive screening tool to investigate and verify latent chemical information of pharmaceutical solid drugs. The developed FTIR/Raman–PCA models present no evidence of drug falsification in 12 paracetamol brands but correlate similar brands based on characteristic vibrational and absorption modes of the API (AAP form I).

As technology advances, instruments can generate a large amount of information with multivariate data in a short time. This has led to advanced analysis tools being used to extract as much information as possible. Here, machine learning (ML) has played an important role when traditional chemometrics methods such as PCA yield unsatisfactory results.

ML is a subfield of artificial intelligence (AI) focused on programing computers to learn and improve automatically without explicit instructions. It leverages algorithms that analyze data and use the insights to make predictions and solve problems. ML solves problems in chemistry through the analysis of large datasets generated by analytical instruments and experiments. Models are built to understand the relationships between various chemical variables and to predict important properties or behaviors. Complexity is reduced by extracting relevant information from noisy or overwhelming data, and experimental design is improved while making data analysis more efficient [25,26].

ML is useful when the objective is to classify chemicals according to their brand, thus verifying whether a given product belongs to the manufacturer shown on the label. Some published studies are T. Chao et al. [27] who applied near-infrared (NIR) spectroscopy for the identification of six different brands of detergent powder. Both extreme learning machine (ELM) and its ensemble (EELM) were used for constructing predictive models. A total of 180 samples belonging to six different brands were prepared for the experiment. The model from the EELM algorithm achieved 100% accuracy on both the training and test sets, which was superior to the model from the ELM algorithm.

Z. Hongguang et al. [28] applied near-infrared (NIR) spectroscopy for the rapid classification of five different brands of washing powder. Chemometric calibrations, including partial least square discriminant analysis (PLS-DA), back propagation neural network (BP-NN), and least square support vector machine (LS-SVM), were investigated. Principal components (PCs) were extracted as inputs of BP-NN and LS-SVM models. As for the comparison of the three investigated models, both the BP-NN model and the LS-SVM model successfully classified all samples in the validation set according to their brands. However, the PLS-DA model failed to achieve 100% of classification accuracy.

When IR spectroscopy is used on poorly reflecting or very thick samples, there is inherently a significant loss of light if the source is not tightly focused, as in the case of a Globar, requiring a more complex array of optical components. In addition, the light source must be modulated by an interferometer to enable Fourier transform infrared spectroscopy (FTIR), which reduces the light output. The practical importance of brightness should not be overlooked in optical metrology, especially in spectroscopic measurements. The brightness of a light source directly affects the spectral power incident on the sample unit area. For mid-IR spectroscopy, this means that, according to the Beer–Lambert–Bouguer law, the light–matter interaction path lengths can be extended, preventing total light attenuation and allowing more molecules to be studied. However, these complications are circumvented with an MIR laser [29,30,31].

Quantum cascade laser (QCL) spectroscopy is a technique that uses quantum cascade lasers as a light source for infrared spectroscopy. Quantum cascade lasers are semiconductor lasers that emit in the mid- to far-infrared region of the electromagnetic spectrum. Unlike conventional lasers, which are based on electron-hole recombination, QCLs use intersubband transitions within the conduction band of a semiconductor material [32].

IR spectroscopy using traditional sources can have limited sensitivity for low-concentration impurities and APIs. This is due to the limited penetration depth in thick or highly scattering tablets. In contrast, quantum cascade laser spectroscopy can penetrate deeper into the sample, allowing analysis of API distribution and potential inhomogeneities within the tablet. QCL spectroscopy can offer higher sensitivity, allowing the detection and quantification of trace amounts of substances. This advantage is particularly beneficial when dealing with complex pharmaceutical formulations where overlapping spectra can be problematic [33].

The use of QCL spectroscopy firstly provides enhanced sensitivity and specificity through high intensity and narrow linewidth emission, resulting in laser source brightness several orders of magnitude higher than thermal emitters. This high specificity is essential for distinguishing between similar compounds and accurately identifying molecular species. Second, it provides wavelength tunability for selective and targeted analysis. Third, it gives an improved signal-to-noise ratio due to the QCL’s coherent, high-intensity output and rapid scanning capabilities for real-time monitoring. The mid-infrared spectra can be acquired up to 1000 times faster with the same detected light intensity, the same detector noise level, and without loss of SNR using the tunable quantum cascade laser compared to the FTIR approach using synchrotron or supercontinuum light. Finally, it presents a compact, robust, and efficient design suitable for portable and field applications [30,34].

This research will propose new methodological strategies that, when applied, would contribute to solving problems associated with the identification and classification of chemicals present in different matrices. Having an analytical method based on infrared spectroscopy for the detection of active pharmaceutical ingredients (in our case AAP) combined with ML is of interest to the pharmaceutical industry and law enforcement agencies. It allows easy and fast molecular information to be obtained to determine whether an API is present in a certain pharmaceutical product and whether it belongs to a brand declared on the label. Using the proposed method will make it possible to know whether the drug is present as a desired product or whether it is a counterfeit drug.

## 2. Results and Discussion

### 2.1. Infrared Spectra Analysis of AAP

**Figure 1:**
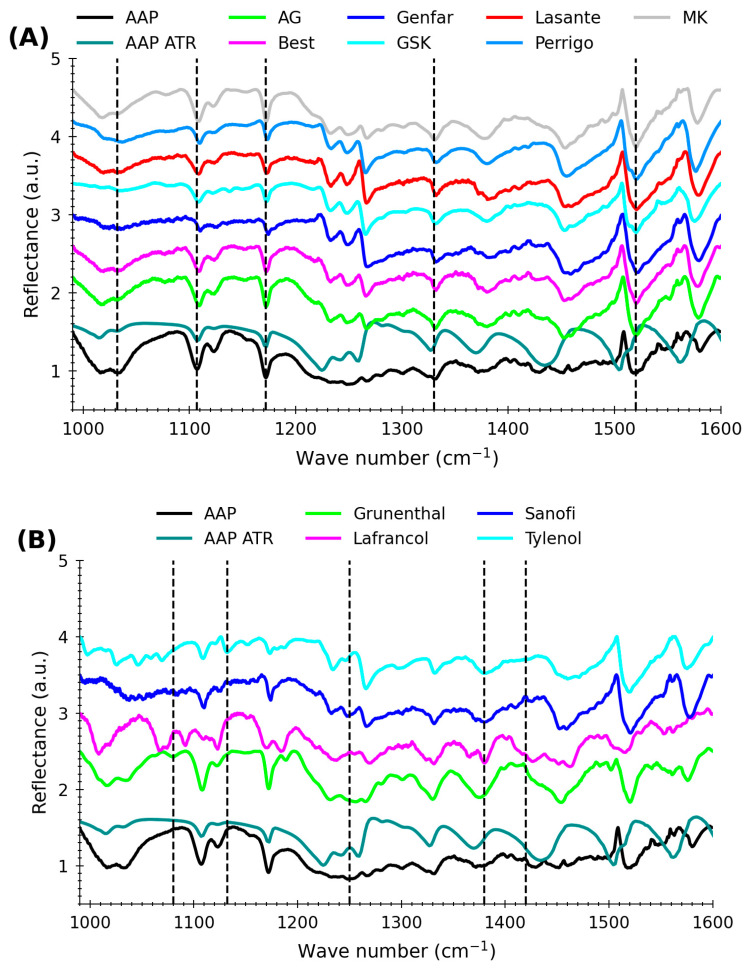
IR spectra of acetaminophen (AAP) using QCL spectroscopy; the vertical dashed lines represent the locations of the main bands. (**A**) Spectra of AAP in different brands containing AAP as the only API. (**B**) Spectra of AAP in different brands containing AAP and other APIs.

Figure 1 shows the spectra of AAP. The spectra were taken using a back reflection geometry employing a LaserScan spectrophotometer. Figure 1A shows the IR spectra of tablets containing AAP as the sole API; additionally, it shows the spectra of standard (pure) AAP using QCL (black spectrum) and ATR-FTIR (red spectrum, in transmittance mode). The ATR-FTIR spectrum was used as a reference spectrum to validate the spectra from QCL. In Figure 1A, it can be observed that the spectra obtained with ATR-FTIR exhibit the characteristic IR vibrational bands of AAP when compared with the FTIR spectra from the literature [23,35,36,37]. Among the most important IR vibrational bands observed in Figure 1A are those for acetaminophen that appear as follows: 1562 cm^−1^ assigned to amide II (N-H in plane deformation), 1520 cm^−1^ for aryl C-H, C-H symmetric bends, 1454 cm^−1^ assigned to skeletal aryl C-C stretch, 1370 cm^−1^ for -CH_3_ (acetamino), 1330 cm^−1^ aligned al CC (phenyl ring), 1260 cm^−1^ and 1210 cm^−1^ related to C-O (phenol), 1170 cm^−1^ associated with C-H (phenyl ring), and 1035 cm^−1^ and 1010 cm^−1^ linked to C-H aromatic.

In the solid formulation of acetaminophen, substances other than APIs are used for various purposes and are called excipients. The excipients commonly used for formulation are lactose, magnesium stearate, starch, and microcrystalline cellulose. These are added in different proportions and vary from product to product. Figure 1B shows mid-infrared spectra of pharmaceutical tablets from different laboratories in the spectral range 990–1600 cm^−1^. These tablets are solid mixtures containing various APIs (as shown in Table 1) and excipients. However, it is noticeable that, for the most part, the IR vibrational spectra depicted in Figure 1A,B are very similar to the IR spectra of standard APIs shown in Figure 1A; therefore, characteristic signals of acetaminophen can be observed in the spectra illustrated in Figure 1A,B.

Visual analysis of the spectra in Figure 1B allows the identification of IR bands in addition to those shown in Figure 1A. These bands belong to the additional APIs found in the solid tablet. Some IR signals of other APIs present are ibuprofen and caffeine (1420 cm^−1^, 1380 cm^−1^, 1360 cm^−1^, 1180 cm^−1^, and 1090 cm^−1^) from Lafrancol [38,39,40], codeine (1264 cm^−1^, 1250 cm^−1^, and 1457 cm^−1^) from Sanofi [41], tramadol (1080 cm^−1^ and 1180 cm^−1^) in Grunenthal [42], and guaifenesin and phenylephrine (1058 cm^−1^, 1069 cm^−1^, 1132 cm^−1^, 1380 cm^−1^, and 1460 cm^−1^) from Tylenol [43]. Additionally, there appears to be no significant interference caused by the excipient. This establishes that the method is practical without carrying out more laborious sample preparation processes such as extraction, thus allowing direct identification of the brand.

### 2.2. Spectral Identification

A useful tool widely used by the analyst for identifying an unknown sample is the use of spectral search. This consists of comparing a spectrum of an unknown sample against a reference spectrum from a database (library). To perform this comparison, each data point in the unknown spectrum is compared to each corresponding point in the reference spectrum. For the spectral search, first, the IR spectrum of the unknown sample is obtained. Next, the obtained spectrum is then compared to spectra in a spectral database. Finally, spectral searching algorithms analyze the fit between an unknown spectrum and the library spectra (by Peak Matching and Peak Intensity). A spectral library (database) is, in essence, a collection of reference spectra for a variety of known materials.

Different mathematical formulas (search algorithms) can be used to compare two spectra point by point. Some of the most common similarity-based search algorithms include Euclidean distance and Pearson correlation coefficient [44]. The spectral correlation coefficient (SCC) is useful for tasks such as compound identification, assessing the quality of spectral data, and comparing experimental results with reference spectra by providing a quantitative measure of the similarity or dissimilarity between spectra. It ranges from −1 to 1, where a value of 1 indicates perfect correlation, meaning that the spectra are identical or very similar. A value of −1 indicates perfect anti-correlation, meaning that the spectra are mirror images of each other, and a value close to 0 indicates little or no correlation between the spectra. The SCC generates a numerical metric (hit quality value—HQV) that indicates how well an unknown material’s spectrum matches a reference spectrum in the spectral library [45].

Table 2 displays the HQV values obtained when comparing the spectrum of a tablet containing AAP from different brands with those from the spectral library using the Pearson correlation coefficient algorithm (*r*) as shown in Equation (1). The spectral library is a collection of different infrared vibrational spectra of AAP tablets of different brands described in Table 1 using QCL spectroscopy. It also contains the spectrum of pure AAP.

(1) $$r=\frac{\left|\sum \left(A_{i}-\bar{A}\right)\left(B_{i}-\bar{B}\right)\right|}{\sqrt{\sum {\left(A_{i}-\bar{A}\right)}^{2}}\sqrt{\sum {\left(B_{i}-\bar{B}\right)}^{2}}}$$

where *A* indicates the library (reference) spectrum and *B* indicates a test spectrum of the samples, *i* denotes the intensity of the *i*th data point (wavenumber), and $\bar{A}$ and $\bar{B}$ designate the average intensity of the *A* and *B* spectra, respectively. To evaluate an unknown spectrum based on its HQV, the following considerations must be taken into account. A high HQV (above 0.9) suggests a good match between the unknown and reference spectra. It is a strong indication that the unknown material is likely the same or very similar to the reference material. A medium HQV (around 0.7–0.9) indicates a good possibility that the unknown material matches the reference, but further confirmation might be needed. Finally, with a low HQV (below 0.7), the match between the unknown and reference spectra is weak [45].

The HQV values shown in Table 2 indicate that the ability to identify the AAP tablet according to its brand depends largely on the composition of the tablet. For brands containing more than one API, brand identification using the spectral library is possible; these have HQV close to 1 (greater than or equal to 0.98). Examples of these are the brands LaFrancol, Grunenthal, and Tylenol. Although the Sanofi brand has another API (Codeine phosphate), the concentration of this (30 mg) is perhaps much lower compared to that of AAP (500 mg). As a result, its HQV (around 0.9) is very similar to other brands containing only AAP, such as Lasante, Best, Genfar, AG, MK, Perrigo, and GSK. When tablets present only AAP with API, according to Table 1, the brand classification by spectral search is much more challenging. Based on an HQV greater than or equal to 0.95 (numbers in red) for spectral identification, Lasente has very similar HQVs to Best, Genfar, and Perrigo. Best is very similar to Lasente, AG, and MK. Genfar can be classified as Lasante, Perrigo, and MK. AG can be branded as Best and MK. MK can be identified as Best and AG. Finally, GSK will be classified as Genfar and Perrigo.

Considering the limited efficiency of spectral identification by the spectral library for the classification of AAPs according to their brands, it is necessary to explore advanced data analysis techniques such as ML.

### 2.3. Machine Learning Analysis

The initial dataset underwent preprocessing to enhance the outcomes. One preprocessing method employed was the first derivative, essential for accentuating specific peaks in the spectrum and achieving improved discrimination between various samples. Another preprocessing technique utilized was the standard normal variance (SNV), which facilitated the normalization of mean values and standardized variance to 1.

#### 2.3.1. Principal Component Analysis

PCA is a powerful technique used in data analysis, particularly for reducing the dimensionality of datasets while preserving crucial information. PCA analyzes the covariance matrix to identify eigenvectors and eigenvalues. Eigenvectors represent the directions of greatest variance in the data, and eigenvalues indicate the amount of variance explained by each eigenvector. The eigenvectors with the highest eigenvalues are chosen as the principal components (PCs). These PCs represent the new axes that capture the most significant information in the data. The original data points are then projected onto these new PCs, creating a lower-dimensional representation of the data.

**Figure 2:**
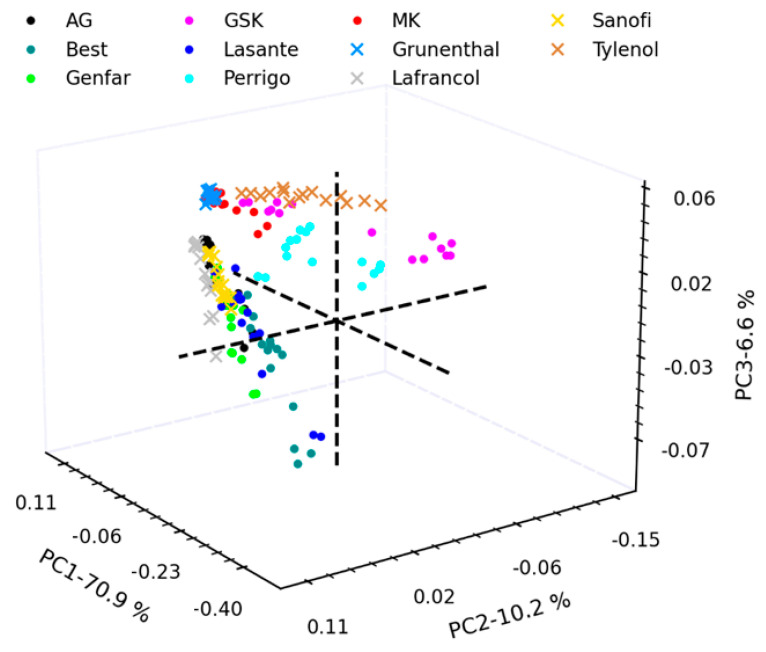
Score plot with 3 principal components using IR spectra of AAP in different brands. The dots marked with a cross represent samples with more than one API, while the dots marked with a circle represent samples with just one API.

Figure 2 shows the score plot. The score plot is a crucial visual representation used in PCA. The score plot displays the data points from the original high-dimensional dataset projected onto the new, lower-dimensional space created by the PCs. Each data point is represented by a dot in the plot, and its co-ordinates correspond to its scores on the chosen PCs. Typically, the first two or three PCs are used for visualization, as they capture the most significant variance in the data [46]. Figure 2 shows that PCA explains 87.7% of the variance. In this diagram, samples containing AAP as the only API are represented by dots, while those with multiple APIs are denoted by crosses. The diagram prominently showcases the grouping of samples from MK, Grunenthal, Perrigo, Lafrancol, and Tylenol laboratories, while the remaining samples are not distinctly separated, rendering them indistinguishable from one another. Additionally, the tested samples can be classified in two ways: firstly, based on their concentration (250 mg, 325 mg, 500 mg, and 650 mg) and, secondly, based on the presence or absence of additional components different from acetaminophen (such as ibuprofen, codeine, tramadol, guaifenesin, and phenylephrine), as shown in Table 1. Upon analyzing the Score diagram, it becomes apparent that the PCA method primarily attempts to classify the samples based on concentration, resulting in the formation of two distinct groups. One group comprises samples with a concentration of 500 mg, located in the grouping’s borders (AG, Best, Genfar, GSK, Lasante, MK, and Sanofis), which happens to be the most prevalent concentration among the samples, while the other group consists of samples with concentrations different than 500 mg located in the center of the grouping (Perrigo, Grunenthal, Lafrancol, and Tylenol). However, this form of classification fails to adequately distinguish the components of the GSK and MK laboratories, as their samples are placed near the group with concentrations different from 500 mg. Moreover, samples with multiple APIs tend to be clustered by the PCA model with three PCs, as observed with the well-grouped brands Sanofi, Lafrancol, Grunenthal, and Tylenol.

Similar to the results obtained for spectral identification, distinguishing between samples from different laboratories, even when the samples only contain acetaminophen, is not achievable.

#### 2.3.2. Analysis of Machine Learning Using SVC, DTC, and ANN

Considering the limited results when spectral search and PCA are used in the identification of AAP tablets by brands, more robust machine learning analyses were considered. Machine learning techniques such as SVC, DTC, and ANN were used to evaluate the capability of these unsupervised learning algorithms. This algorithm analyzes unlabeled data, identifying patterns and structures within the data themselves, to achieve high levels of accuracy in differentiating between tablets containing AAP as a single API from different brands.

An important step in ML is the setting of hyperparameters. The performance of ML models is directly influenced by hyperparameters. Choosing the right hyperparameters can have a significant impact on the effectiveness, efficiency, and robustness of the models deployed. This setting is unique for each analysis you wish to perform and depends on the specific dataset and its characteristics, the type of machine learning algorithm used, and the desired performance metrics [26,47,48,49].

Table 3 presents the cross-validation results for each method, illustrating the examined values for every hyperparameter. Subsequent results will be obtained by configuring each method with the values specified in Table 3. This sequential approach ensures a thorough exploration of hyperparameter settings, thereby enhancing the understanding of the model’s performance.

A useful tool for evaluating the effectiveness of classification models in ML is the confusion matrix (CM). A CM is a table used to evaluate the performance of a classification algorithm in machine learning. Each cell of the table shows the number of data points that belong to a specific combination of actual and predicted classes. The performance of a model can be visualized by comparing predicted classes with true classes. The confusion matrix provides a summary of the number of correct and incorrect predictions made by the model from SVC, DTC, and ANN [25,47].

**Figure 3:**
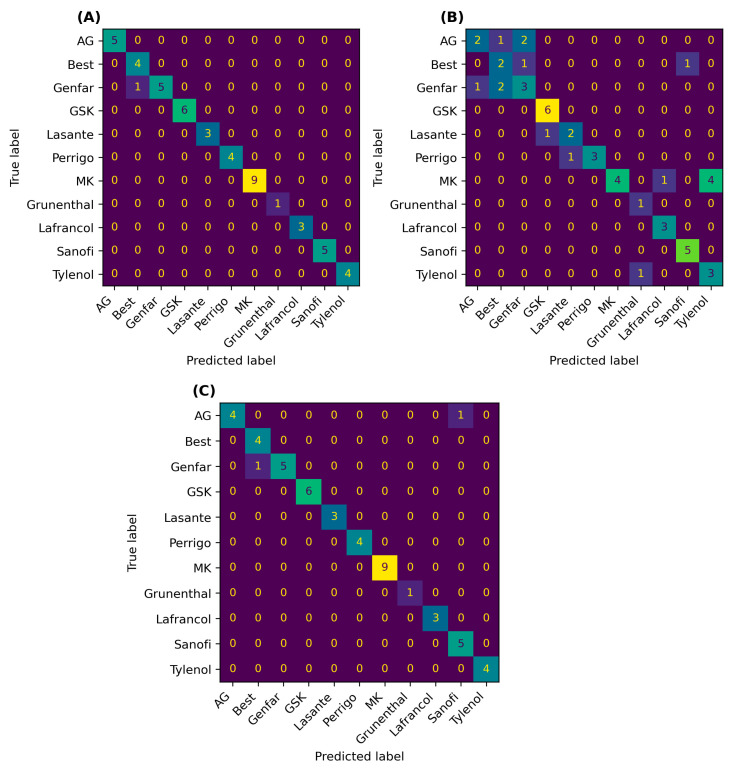
Confusion matrices for the test data in the classification of (**A**) SVC, (**B**) DTC, and (**C**) ANN.

In Figure 3, the confusion matrix is depicted, offering a straightforward and visual method to evaluate the performance for each class among SVC, DTC, and ANN methods. It is evident from this matrix that SVC showcases the most robust performance, with only 1 error detected out of 50 test samples. ANN also presents promising results, with a mere two errors. However, the performance of DTC was comparatively less favorable, with 16 errors identified.

Using ML algorithms such as SVC and ANN, it is possible to differentiate between brands with AAP as the only API. These results overcome the limitations encountered when spectral search and PCA are used as tools to identify AAP tables by brands. Across all three ML algorithms, distinguishing between samples from BEST and Genfar laboratories proves to be somewhat challenging. However, when SVC and ANN are employed, these brands can be identified with high accuracy when compared to DTC. An error for ANN occurred between a sample with mere acetaminophen (AG) and another with additional components (Sanofis). Notably, Sanofis contains APP 500 mg and is the brand with the sample containing a lower concentration of additional API (30 mg of codeine phosphate). Probably Sanofi, having the same concentration of AAP as AG, presents spectral similarity as indicated by the HQV values in Table 2. In the case of DTC, most errors resulted from misclassifying samples containing only acetaminophen (9 out of 16 errors). Overall, all three methods effectively differentiate between samples with acetaminophen as a single API and those with additional components. This underscores the efficacy of machine learning methods for detecting pharmaceutical samples according to their manufacturer.

To evaluate the model’s performance, precision (*Pr*), recall (*Rc*), specificity (*Es*), F1-score (*Fs*), and accuracy (*Ac*) metrics were employed. These metrics, commonly utilized for assessing classification problems, have been demonstrated in various studies [48,49,50]. They are defined in Equations (2)–(6), where true positive (TP) signifies instances where the model correctly identifies positive outcomes, while true negative (TN) denotes correct identification of negative outcomes. False positive (FP) indicates instances where the model erroneously identifies a negative outcome as positive, and false negative (FN) represents erroneous identification of a positive outcome as negative. Through a comprehensive analysis of these metrics within the confusion matrix, the model’s performance and predictive accuracy can be thoroughly evaluated.

(2) $$Pr=\frac{TP}{TP+FP}$$

(3) $$Rc=\frac{TP}{TP+FN}$$

(4) $$Es=\frac{TN}{TN+FP}$$

(5) $$Fs=\frac{2\times Pr\times Rc}{Pr+Rc}$$

(6) $$Ac=\frac{TP+TN}{TP+TN+FP+FN}$$

The *Pr* assesses the accuracy of positive predictions, while Rc measures the model’s ability to capture all relevant instances. Es gauges the model’s ability to correctly identify negative instances. Fs is the harmonic mean of precision and offers a balanced assessment. The Ac is the ratio of correctly predicted instances to the total instances, providing an overall measure of the model’s correctness.

**Figure 4:**
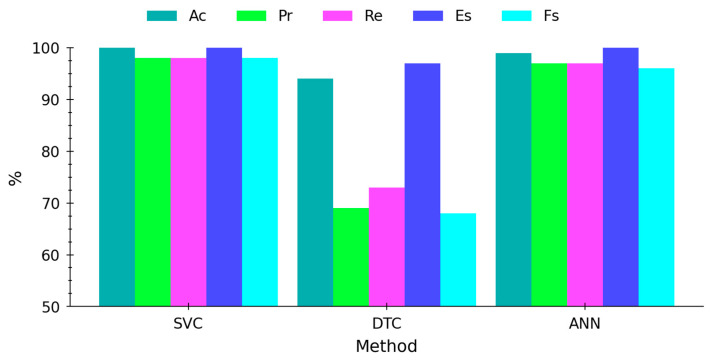
Average performance metrics for classification models’ predictive testing.

In Figure 4, the averages of the main metrics used to evaluate the performance of the classification problem are illustrated. Remarkable accuracy has been achieved in classifying acetaminophen samples. Both support vector classification (SVC) and artificial neural network (ANN) methods have demonstrated high effectiveness, with precision, recall, and F1-score metrics generally either perfect or near perfection. Remarkably, the accuracy (Ac) for all three methods exceeds 94%, underscoring the robustness of the classification results (see Figure 4). Although decision tree classification (DTC) exhibits slightly lower performance compared to SVC and ANN, it still maintains a notable capability to classify samples accurately. These findings collectively suggest that machine learning models can effectively distinguish between acetaminophen samples, holding significant implications for ensuring product quality and authenticity within the pharmaceutical industry and of interest to law enforcement agencies.

## 3. Materials and Methods

To create our sensor based on mid-infrared spectroscopic data and ML for direct detection and classification of acetaminophen tablets according to their brand, the following methodology was used.

### 3.1. Sample and Standard Acquisition

The reagents used in this study correspond to standards and samples. Pure analytical-grade acetaminophen purchased from Sigma-Aldrich (Milwaukee, WI, USA) was used as a standard to obtain the reference mid-infrared vibrational spectrum of AAP. Solid tablet formulations containing AAP as API were used as samples. These samples were obtained from local drugstores in Cartagena–Colombia, Puerto Rico, and Mexico. The acquired samples containing AAP were from different manufacturing laboratories (brands) as shown in Table 1. In total, 15 tablets of AAP were purchased by brand, for a total of 165 samples in the 11 brands tested. These were purchased from different drugstores and dates to ensure different batch manufacturing dates.

### 3.2. Sample Preparation

No wet chemistry was used for sample preparation in this study. The only step involved was the pulverization of a small portion of the solid pharmaceutical samples in a mortar to reduce the particle size of the sample. If the tablet had a coating, it was removed before pulverizing.

### 3.3. Acquisition of Spectra

**Figure 5:**
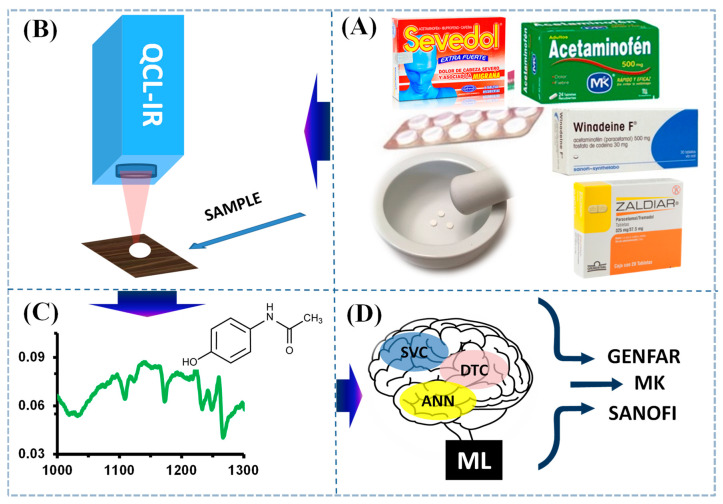
Experimental setup: analyte detection using spectroscopy. (**A**) Sample preparation, (**B**) QCL detection, (**C**) spectral analysis, and (**D**) ML analysis.

Spectroscopy based on quantum cascade lasers (QCL) was used to obtain the mid-infrared (MIR) vibrational spectra of AAP. Infrared spectra of AAP from commercial tablets, as well as spectra taken from the solid AAP standard were recorded using the spectrometer LaserScan^TM^ from Block Engineering (900–1600 cm^−1^, Marlborough, MA, USA), 4 mm × 2 mm beam, thermoelectrically cooled Hg-Cd-Te (MCT) detector. The system was designed to operate in back reflection mode (at zero angle concerning the surface normal, as shown in Figure 5B.

MIR spectra were recorded at a distance of 6 in. from pulverized sample at 4 cm^−1^ resolution and 2 Co-add. The background spectra were obtained using flat and clean Al substrates (31 mm × 31 mm). One spectrum was taken for each sample, for a total of 15 spectra per brand. Thus, 165 independent spectra were obtained. All spectra were stored in Thermo-Galactic™ SPC format (Thermo Fisher Scientific, Inc., Waltham, MA, USA).

### 3.4. Machine Learning Analysis

The spectra obtained were subjected to machine learning analysis such as principal component analysis (PCA), support vector classification (SVC), decision tree classifier (DTC), and artificial neural network (ANN) with several preprocessing steps. Both data processing and preprocessing stages leveraged the scikit-learn machine learning library for Python [51]. The library’s StandardScaler function was instrumental for SNV, implementing a mean centering algorithm during preprocessing. Consequently, the data were rescaled by subtracting the mean and dividing by the standard deviation [25].

Table 4 presents the chosen hyperparameters for the hyperparameter optimization of SVC, DTC, and ANN models. The selected hyperparameter values were determined based on commonly used settings in similar investigations [52,53]. The ultimate optimal values for each hyperparameter were determined through cross-validation, employing a grid search approach.

The entire dataset underwent a random splitting strategy, allocating 70% for training and reserving the remaining 30% for testing purposes. During the cross-validation process, the samples were further partitioned into 5 groups using the KFold method. In KFold cross-validation, the dataset is divided into ‘k’ folds or subsets, and the model is trained ‘k’ times, each time using a different fold as the testing set and the remaining folds as the training set. This iterative process ensures a comprehensive evaluation of the model’s performance across different subsets of the data, promoting robustness and reliability in the assessment.

Figure 5 shows the experimental setup used in this research, summarizing the steps performed in this research: (Figure 5A) Step I, sample selection and preparation; (Figure 5B) Step II, QCL detection; (Figure 5C) Step III, vibrational spectra; and (Figure 5D) Step IV, classification by ML.

## 4. Conclusions

This study successfully developed a method for identifying and classifying acetaminophen tablets according to their brand using QCL-based mid-infrared spectroscopy and machine learning. The proposed methodology offers a promising solution for quality control and brand authentication in the pharmaceutical industry and law enforcement agencies for counterfeit detection. Spectral identification using a spectral library showed limitations in differentiating between brands, particularly for tablets containing AAP as the single API. PCA analysis was able to partially group samples based on concentration but struggled to distinguish between brands of AAP tablets containing only AAP. Machine learning models, specifically SVC and ANN, achieved high accuracy in classifying AAP tablets according to their brand, even for brands containing mere AAP. Confusion matrix analysis revealed that SVC had the most robust performance with only one error, followed by ANN with two errors. DTC exhibited lower accuracy with 16 errors but it effectively differentiated between samples with and without additional APIs. This research demonstrates the effectiveness of ML models in combination with mid-infrared spectroscopy for brand identification of AAP tablets. Using this approach to distinguish between brands offers significant advantages for quality control, brand authentication, and potentially law enforcement applications. Further research could explore the extension of this methodology to other pharmaceutical products and brands, as well as the integration of additional analytical techniques to enhance classification accuracy.

## Figures and Tables

**Table 1:** Composition of tablet of AAP according to its commercial brand.

Country	APIs Present	Brand
Colombia	Acetaminophen 500 mg	AG
Colombia	Acetaminophen 500 mg	Best
Colombia	Acetaminophen 500 mg	Genfar
Puerto Rico	Acetaminophen 500 mg	GSK
Colombia	Acetaminophen 500 mg	La Sante
Mexico	Acetaminophen 650 mg	Perrigo
Colombia	Acetaminophen 500 mg	MK
Colombia	Acetaminophen 325 mg	Grunenthal
	Tramadol 37.5 mg	
Colombia	Acetaminophen 250 mg	La Francol
	Ibuprofen 400 mg	
	Caffeine 65 mg	
Colombia	Acetaminophen 500 mg	Sanofis
	Codeine phosphate 30 mg	
Puerto Rico	Acetaminofén 325 mg	Tylenol
	Guaifenesin 200 mg	
	Phenylephrine HCl 5 mg	

**Table 2:** HQV values for AAP spectra of different brands using the Pearson correlation coefficient algorithm.

Tylenol	Sanofi	Lafrancol	Grunenthal	MK	Perrigo	Lasante	GSK	Genfar	Best	AG	Spectral Library
0.89	0.91	0.50	0.66	0.89	0.96	0.98	0.93	0.96	0.97	0.93	Lasante
0.90	0.93	0.59	0.79	0.96	0.95	0.97	0.93	0.92	0.97	0.98	Best
0.87	0.90	0.47	0.57	0.85	0.95	0.96	0.95	0.94	0.92	0.89	Genfar
0.82	0.91	0.67	0.84	0.99	0.89	0.93	0.91	0.89	0.98	0.99	AG
0.89	0.91	0.56	0.73	0.90	0.94	0.91	0.93	0.90	0.93	0.91	Sanofi
0.45	0.56	0.99	0.71	0.70	0.46	0.50	0.53	0.47	0.59	0.67	Lafrancol
0.81	0.90	0.70	0.89	0.99	0.87	0.89	0.89	0.85	0.96	0.99	MK
0.63	0.73	0.71	0.99	0.89	0.66	0.66	0.71	0.57	0.79	0.84	Grunenthal
0.93	0.94	0.46	0.66	0.87	0.99	0.96	0.96	0.95	0.95	0.89	Perrigo
0.87	0.93	0.53	0.71	0.89	0.96	0.93	0.99	0.95	0.93	0.91	GSK
0.98	0.89	0.45	0.63	0.81	0.93	0.89	0.87	0.87	0.90	0.82	Tylenol

**Table 3:** Hyperparameter values resulting from cross-validation for each method.

Values	Hyperparameters	Method
1.023292	C	SVC
0	Γ	
Lineal	Kernel	
7	Max_depth	DTC
5	Min_samples_split	
log_loss	Criterion	
20	Neurons	ANN
1	Hidden layers	
lbfgs	Solver	

**Table 4:** Hyperparameter values tested for cross-validation of ML models.

	Hyperparameters	Method
$10^{x}$, x∈ [0.01; …; 2] [0; 0.07; …; 1] × 10^2^	C	SVC
	Γ	
[‘linear’; ‘Rbf’; ‘Sigmoid’]	Kernel	
[3; 5; 7; 10][2; 5; 10]	Max_depth	DTC
	Min_samples_split	
[‘Gini’; ‘Entropy’; ‘Log_loss’]	Criterion	
[5; 10; 20]	Neurons	ANN
[1; 2; 3]	Hidden layers	
[‘Lbfgs’; ‘Sgd’; ‘Adam’]	Solver	

## Data Availability

Dataset available on request from the authors.
